# The Effects of Mindfulness Techniques on Anxiety, Depression, and Stress, with an Emphasis on Gratitude: A Systematic Review and Meta-Analysis

**DOI:** 10.3390/healthcare14050601

**Published:** 2026-02-27

**Authors:** Mădălina Sarca, Adriana Cojocaru, Raluca Dumache, Brenda Cristiana Bernad, Laura Alexandra Nussbaum, Iuliana Costea, Teodora Anghel, Lavinia Hogea

**Affiliations:** 1 Doctoral School of Medicine, “Victor Babes” University of Medicine and Pharmacy, 300041 Timisoara, Romania; madalina.sarca@umft.ro; 2 Neuroscience Department, “Victor Babes” University of Medicine and Pharmacy, 300041 Timisoara, Romania; bernad.brenda@umft.ro (B.C.B.); nussbaum.laura@umft.ro (L.A.N.); anghel.teodora@umft.ro (T.A.); hogea.lavinia@umft.ro (L.H.); 3 Neuropsychology and Behavioral Medicine Center, “Victor Babes” University of Medicine and Pharmacy, 300041 Timisoara, Romania; 4 Psychology Department, West University of Timisoara, 300223 Timisoara, Romania; iuliana.costea@e-uvt.ro

**Keywords:** mindfulness, gratitude, anxiety, depression, stress, mindfulness-based interventions, mental health, well-being, systematic review, meta-analysis

## Abstract

**Background/Objectives:** Mental health conditions such as anxiety, depression, and stress remain among the leading global causes of disability. Mindfulness-Based Interventions (MBIs) have gained increasing attention as effective non-pharmacological strategies for reducing psychological distress. **Methods:** This systematic review examined 30 randomized controlled trials and quasi-experimental studies involving over 24,000 participants to evaluate the impact of MBIs on mental health outcomes, with a specific focus on the contribution of gratitude-based components. **Results:** Studies varied in terms of population, duration, and format, with most demonstrating moderate to strong effects on symptom reduction, particularly in programs lasting 8 to 12 weeks. A random-effects meta-analysis was conducted, yielding a pooled effect size of Hedges’ g = −0.45, indicating a moderate improvement in psychological outcomes. Subgroup analyses revealed slightly stronger effects for anxiety (g = −0.56) than depression (g = −0.45). Gratitude-integrated MBIs demonstrated modestly enhanced emotional benefits, suggesting a synergistic role in improving well-being. **Conclusions:** The review found low evidence of publication bias and acceptable risk of bias, supporting the moderate results. The findings underscore the value of MBIs, particularly those integrating gratitude, as scalable, cost-effective interventions in clinical and educational settings.

## 1. Introduction

The global burden of anxiety, depression, and stress has grown considerably, driven by complex sociocultural, economic, and environmental factors. Around the world, in 2019, mental health conditions were widespread and rising; an estimated 28% of the global population experienced depression and about 26.9% suffered anxiety, while stress affected roughly 36.5% [1,2]. Anxiety disorders alone affect around 4% of people globally—over 300 million individuals—and have risen sharply (a more than 55% increase since 1990). Depression is likewise a leading contributor to global disability, impacting some 4.4% of the population, with its burden growing by nearly 18% between 2005 and 2015 [3,4].

Mental health outcomes also differ by gender. Research shows women tend to report higher levels of anxiety and depression compared to men [5,6]. Among students, female medical students often experience more significant psychological decline than their male peers [7,8]. The burden of mental health disorders underscores the urgent need for accessible, scalable, and non-invasive mental health interventions to complement traditional care approaches [9,10].

Mindfulness, with its roots in ancient Indian philosophy, is a mental discipline focused on maintaining non-judgmental awareness and attention to the present moment. It draws from traditional ideas such as “Sakshi” (the observer self) and “Dhyana” (focused meditation) [11]. In recent years, mindfulness-based therapies (MBTs) have emerged as effective treatments for mental health conditions like anxiety, depression, and stress [12,13]. Extensive research confirms the benefits of mindfulness interventions in self-directed approaches, which have shown notable reductions in psychological symptoms across varied groups, including individuals managing chronic diseases such as cancer [14].

Gratitude is a complex and well-studied emotion within psychology, widely acknowledged for its role in fostering interpersonal relationships and promoting overall well-being. Defined as the recognition and appreciation of benefits received from others, gratitude strengthens social cohesion. It is viewed in positive psychology as a character strength that bolsters resilience and mental health [15,16]. The broaden-and-build theory suggests that gratitude expands individuals’ cognitive and emotional resources, promoting psychological resilience and social connectedness [17]. Empirical evidence highlights the benefits of gratitude in enhancing life satisfaction, reducing anxiety and depression, and fostering stronger social ties [18,19].

The integration of mindfulness and gratitude represents a promising strategy for enhancing psychological well-being, as studies have demonstrated that these two constructs mutually reinforce each other [20]. When combined, mindfulness and gratitude practices can produce synergistic benefits, including improved emotional regulation, reduced anxiety, and increased life satisfaction. Intervention models often incorporate practices like gratitude journaling alongside mindfulness meditation to deepen emotional insight and support mental health [21].

Despite growing evidence supporting the effectiveness of MBIs in reducing symptoms of anxiety, depression, and stress, few studies have systematically examined the role of gratitude as a complementary or enhancing component within these interventions. While gratitude practices have independently shown promise in promoting psychological well-being, their integration into mindfulness protocols remains underexplored. Existing research often treats mindfulness and gratitude as separate constructs, with limited investigation into their potential synergistic effects when combined. There is a lack of comprehensive reviews that synthesize findings on this integrated approach, highlighting the need for a systematic evaluation of the evidence. Addressing these gaps could inform more holistic and effective interventions for mental health promotion.

Given the increasing prevalence of anxiety, depression, and stress worldwide, there is a growing demand for accessible and evidence-based psychological interventions. Mindfulness-based practices have gained empirical support for their effectiveness in improving mental health outcomes, and gratitude has emerged as a promising construct in positive psychology.

This review aims to systematically assess the effects of MBIs, particularly those incorporating gratitude, on anxiety, depression, and stress. By synthesizing existing studies, this review aims to clarify whether integrating gratitude into mindfulness enhances intervention outcomes, identify gaps in current evidence, and inform future clinical practice and research. The findings may contribute to the development of more comprehensive, cost-effective, and emotionally enriching interventions.

## 2. Materials and Methods

The primary objective of this study was to evaluate the effects of mindfulness-based interventions, including gratitude components, on depression, anxiety, and stress outcomes. We hypothesized that these interventions would be associated with significant improvements in mental health outcomes compared with control conditions.

### 2.1. Search Strategy

This systematic review was conducted following the PRISMA (Preferred Reporting Items for Systematic Reviews and Meta-Analyses) guidelines [22]. The review protocol was developed a priori and registered in the International Prospective Register of Systematic Reviews (PROSPERO) under the registration number: CRD420251276109. Artificial intelligence tools, including language models (ChatGPT GPT-5.2, https://chat.openai.com accessed on 2 January 2026 and ElicitAI, https://elicit.com accessed on 26 December 2025), were used to support the formulation of the study design, assist with literature screening strategies, and aid in structuring the data analysis. AI-assisted tools were used exclusively to support the literature screening and organization process (e.g., duplicate detection and reference management), while all eligibility decisions, data extraction, and analyses were performed manually by the authors. All outputs were critically reviewed and validated by the authors to ensure accuracy, academic integrity, and methodological rigor.

### 2.2. Study Selection

The literature search was conducted using the Semantic Scholar, PubMed, and PsycInfo corpora via the Elicit platform. Studies were eligible for inclusion if they (1) involved participants aged 12 or older; (2) implemented multi-session MBIs (standard or gratitude-enhanced); (3) used a randomized controlled trial or quasi-experimental design with a control group; (4) included at least one validated measure of anxiety, depression, or stress; and (5) employed quantitative data analysis. Only studies published within the last 10 years were included to capture contemporary mindfulness-based intervention protocols and outcome assessment methods. Studies were excluded if they (1) did not employ a randomized controlled or quasi-experimental design; (2) lacked a control or comparison group; (3) did not report validated quantitative measures of anxiety, depression, or stress; (4) involved single-session or purely qualitative interventions; (5) focused exclusively on populations younger than 12 years; or (6) provided insufficient data to allow outcome extraction.

**Figure 1:**
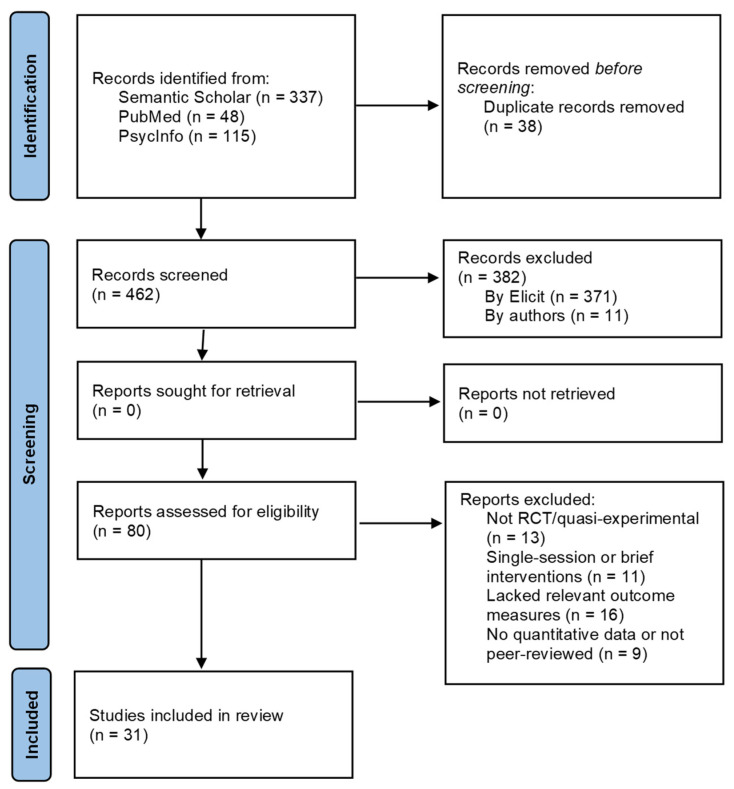
Study selection process in accordance with the PRISMA guidelines.

The literature search was conducted using the Elicit platform, employing predefined keywords combined with Boolean operators. The primary search string included the following terms: (“mindfulness” OR “mindfulness-based intervention”) AND (“gratitude”) AND (“anxiety” OR “depression” OR “stress” OR “mental health”). The database search returned 500 records, which were all screened independently by two authors at the title and abstract levels using predefined inclusion criteria. Title and abstract screening were performed independently by two authors. Full-text eligibility assessment and data extraction were also conducted independently by the same reviewers using a predefined extraction template. Any discrepancies were resolved through discussion, and when consensus could not be reached, a third author was consulted. Artificial intelligence tools (Elicit AI and large language models) were used to assist in literature screening and structured data extraction; however, all AI-generated outputs were manually verified and validated by the authors prior to inclusion. The study selection process is illustrated in Figure 1, following PRISMA guidelines.

### 2.3. Data Items and Data Analysis

For the purpose of this review, gratitude-integrated MBIs were defined as interventions that explicitly incorporated structured gratitude practices (e.g., gratitude journaling, reflective gratitude exercises, or compassion-focused gratitude components) into the intervention protocol.

The following data items were extracted from each included study: (1) Study design, specifying whether the methodology was a randomized controlled trial, quasi-experimental design, or meta-analysis; (2) Participant characteristics, including sample size, age range or mean age, gender distribution, population type (e.g., students, clinical, general population), and baseline mental health status; (3) Intervention details, such as the type of MBIs (e.g., MBSR, MBCT, web-based), delivery method (group, individual, digital), duration and frequency of sessions, and presence of any gratitude components; (4) Primary outcome measures, including the specific mental health domains assessed (e.g., anxiety, depression, stress), measurement instruments used (e.g., Beck Depression Inventory), and time points of data collection; (5) Key findings, including effect sizes, confidence intervals, *p*-values, and statistical significance of results; and (6) Study setting and contextual factors, including geographic location, type of institution (e.g., university, clinical center), and any relevant environmental conditions. Where applicable, outcome maintenance over time and subgroup-specific effects were also noted.

### 2.4. Risk of Bias

Risk of bias was assessed using the Cochrane RoB 2.0 tool across five predefined domains (randomization process, deviations from intended interventions, missing outcome data, measurement of outcomes, and selection of the reported results), with studies categorized as low risk, some concerns, or high risk according to Cochrane guidance.

For studies included in the review, effect measures were extracted and reported according to the statistical analyses provided. These included standardized mean differences (Hedges’ g), risk ratios, and other relevant statistics such as *p*-values, confidence intervals, and percentage change. When available, effect sizes were interpreted in the context of clinical relevance, with particular attention to outcomes related to anxiety, depression, and stress. For studies that did not report effect sizes explicitly, available data were used to estimate or qualitatively interpret the intervention’s impact.

The strength of evidence for each primary outcome—anxiety, depression, and stress—was rated as high, moderate, low, or very low depending on the quality and coherence of the findings across included studies.

### 2.5. Statistical Analysis

The analyses were conducted using Python (v3.11) and the Statsmodels library (v0.14.0), complemented by Matplotlib (v3.8.4) for visualizations. A random-effects meta-analysis was performed using the DerSimonian–Laird method to calculate the pooled effect sizes (Hedges’ g) and their associated standard errors. Heterogeneity was examined by estimating the variance among effect sizes. To assess potential publication bias, Egger’s regression test was applied to detect funnel plot asymmetry. Subgroup analyses were conducted separately for depression and anxiety outcomes. All statistical analyses were performed by the authors using predefined scripts, and all outputs were independently checked for consistency and accuracy, as outlined in the PRISMA and Cochrane recommendations.

## 3. Results

### 3.1. Overview of Selected Studies

The initial database search, conducted with predefined keywords, yielded 500 records. Following a preliminary title and abstract screening, 38 duplicate entries and 382 irrelevant records—mainly due to unrelated aims—were excluded. This left 80 full-text articles for detailed assessment. During the full-text review, 43 studies were excluded for various reasons, including a lack of alignment with the research objectives, insufficient reporting of outcomes, the absence of validated measures, or the use of non-eligible study designs (e.g., single-session interventions or purely qualitative studies). Ultimately, 30 studies met all inclusion criteria and were incorporated into the final synthesis (Figure 1). Details regarding the excluded studies and reasons for exclusion are available upon request or in Supplementary Materials (Table S1).

The included studies span 13 countries, reflecting a diverse global interest in mindfulness-based interventions (MBIs). The United States contributed the most studies (*n* = 9), followed by China (*n* = 5), Spain (*n* = 4), Brazil (*n* = 3), and Finland (*n* = 2). Other countries represented include Canada, India, Sweden, the UK, Turkey, Jordan, Switzerland, and Australia. This geographical spread enhances the generalizability of the findings while highlighting cultural variability in implementation and outcomes.

The included studies span a diverse range of countries, with a strong representation from the United States [23,24,25,26,27,28,29,30] and multiple studies from China [31,32,33,34,35]. Other represented countries include Canada [24,36], Brazil [37,38,39], Spain [40,41,42], Finland [43,44], the UK [45,46], Sweden [47], Turkey [48], India [49], Jordan [50], and Switzerland [51].

In terms of study design, the vast majority of the research employed randomized controlled trials (RCTs), thereby enhancing the validity and reliability of the findings. Only a few studies used quasi-experimental designs [41,49].

Regarding populations, there was wide variation. Several studies targeted university students [24,39,45,46]. In contrast, others focused on adolescents [26,30,31,34,40,51], older adults [25,36], and clinical populations including individuals with anxiety, depression, or hypertension [27,28,29,33]. Some studies also included at-risk or high-stress professionals such as police officers [37], nurses [50], and healthcare workers [42].

Most studies have implemented group-based mindfulness interventions, including standard protocols such as Mindfulness-Based Stress Reduction (MBSR) and Mindfulness-Based Cognitive Therapy (MBCT). For instance, Chi et al. used MBSR with adolescents and young adults, while Torres-Platas et al. applied MBCT for older adults in Canada [31,36]. Some studies explored adapted or novel programs, such as the M-Body program [29], Mindful Self-Compassion for Teens [26], and Learning to BREATHE [52]. Digital delivery was also prominent in several studies [24,32,35,46,48], reflecting the growing interest in online and mobile mindfulness formats.

In terms of outcomes, the most frequently measured variables were depression, anxiety, and stress, often assessed using validated psychometric instruments. Several studies also explored secondary outcomes, including self-compassion [26], resilience [43], insomnia [39], biomarkers [28], and functional brain changes via fMRI [51].

Effect sizes and statistical significance were reported in most studies, showing generally positive and significant improvements in mental health outcomes. For instance, El Morr et al. found large effect sizes for both depression and anxiety (Cohen’s d = −0.69/−0.74) [24], while Torres-Platas et al. also reported significant effects (d = 0.86/0.99, *p* < 0.01) [36]. Moderate effect sizes were observed by Chi et al. for depression (Hedges’ g = −0.45) [31] and Shomaker et al. for depression in adolescent girls (d = 0.68, *p* = 0.03) [52]. Some studies, such as those by Galante et al., reported strong effects compared to no intervention, but not when compared to active controls [23]. A few studies, such as Sundquist et al., found improvements within groups but no significant differences between groups [47].

The duration of the interventions varied, with many programs lasting 8 weeks [24,25,34,40,48]. Others were shorter—such as the 3–6-week programs by Antony and Prasad (India) [49], Wang (China) [35], and Freedenberg (US) [30]—or longer, such as the 36-week duration for Bluth et al., which included follow-up sessions [26]. The duration of the intervention appeared to influence the magnitude and sustainability of outcomes, with programs lasting 8 to 12 weeks consistently yielding moderate to considerable improvements in depression and anxiety. In comparison, shorter interventions (3–4 weeks) showed positive but more limited effects, and longer formats (e.g., 36 weeks) tended to support greater long-term impact, especially on resilience and emotion regulation, Table 1.

A total of 24,563 participants were included across the 30 studies that reported sample sizes. The minimum sample size was 32 participants [52], while the maximum was 11,605 participants in the large-scale study [23]. The median number of participants was 112, and the mean sample size across all studies was approximately 818.8, skewed mainly by the two extensive samples reported by Galante et al. (11,605) and Volanen et al. (3519, reported twice in separate studies from 2020 and 2024) [23,43,44]. When excluding these outliers, the adjusted mean was approximately 160 participants per study, which is more typical of trial sizes. The 25th percentile of sample size distribution was 59 and the 75th percentile was 215, showing that half of the studies had between 59 and 215 participants. Most studies had moderate sample sizes, supporting the robustness of the findings while highlighting the diversity in study scale across international contexts.

### 3.2. Risk of Bias Assessment

All 30 randomized controlled trials (RCTs) included in this systematic review were assessed using the revised Cochrane Risk of Bias 2.0 tool (RoB 2.0) across five domains (Table S2, available in the Supplementary Materials). The overall quality of evidence was generally acceptable, with most studies categorized as having low to moderate risk of bias. However, several studies displayed methodological limitations that warrant careful consideration.

Looking at the randomization process, most trials adequately described or implemented random allocation. For instance, Galante et al. (2021), Hoge et al. (2022), and Wang et al. (2023) detailed appropriate random sequence generation and allocation concealment and were judged to be at low risk in this domain [23,27,35]. In contrast, studies such as those by Chi et al. (2018) and El Morr et al. (2020) provided insufficient detail regarding the randomization process, leading to a classification of “some concerns” [24,31]. Blinding was not feasible in many studies because the interventions were behavioral. While most studies mitigated this limitation by standardizing procedures and training facilitators, moderate risk was identified in several trials [46,47]. However, studies such as those by Burnett-Zeigler et al. (2023) and Zhang et al. (2024) demonstrated a low risk by maintaining consistent delivery formats and minimizing protocol deviations [29,33].

Attrition was generally low across the included studies, with 25 out of 30 trials rated as low risk in missing outcome data. Nevertheless, moderate risk was noted in studies such as Shomaker et al. (2019) and Alfurjani et al. (2023), primarily due to incomplete follow-up reporting or lack of imputation strategies [50,52].

For measurement of the outcome, given the reliance on self-reported psychological measures (depression, anxiety), blinding of outcome assessors was rarely reported. Despite this, most studies used validated instruments, reducing potential detection bias. Studies such as Gallo et al. (2023) and Freedenberg et al. (2016) were rated as having a moderate risk due to unblinded measurement and a lack of corroborating objective data [30,39]. The risk of selective outcome reporting was generally low. However, studies lacking pre-registered protocols [40,41] were rated as moderate to high risk in this domain. The absence of protocol transparency was particularly concerning in the study by Assumpção et al. (2018), which also showed high risk in other domains [38].

Overall, out of the 30 studies, 11 were rated as low risk of bias, 13 as moderate (“some concerns”), and six as high risk. Notably, the study by Burnett-Zeigler et al. (2023), which focused on a mindfulness intervention among underserved women at a Federally Qualified Health Center, achieved low-risk ratings across all five domains, representing one of the most methodologically robust trials in the review [29].

### 3.3. Statistical Analysis Results

A quantitative synthesis was conducted using effect sizes (Cohen’s d, Hedges’ g) reported across the included studies (*n* = 30) (Data table available in Supplementary Materials, Table S3). Where sufficient data were available, a random-effects meta-analysis was planned to estimate the pooled effect of MBIs on outcomes such as depression, anxiety, and stress. Heterogeneity was assessed using the I^2^ statistic, and potential publication bias was evaluated through funnel plots and Egger’s test. Subgroup analyses were considered based on delivery format, population type, and inclusion of gratitude components.

**Figure 2:**
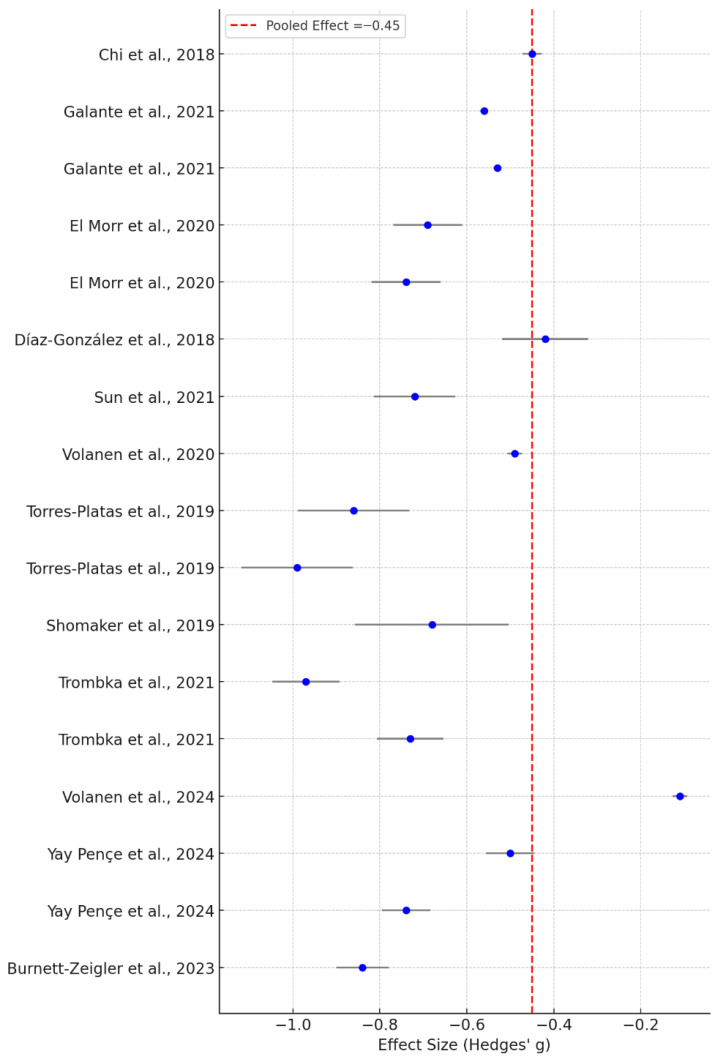
Forest plot showing individual and pooled effect sizes (Hedges’ g) for the effect of mindfulness-based interventions on depressive symptoms. Points represent study-specific effect sizes and horizontal lines indicate 95% confidence intervals; the dashed vertical line represents the pooled effect estimate. Negative effect sizes indicate a reduction in depressive symptom severity, favoring the intervention group compared to controls, whereas positive effect sizes indicate higher symptom levels or less improvement in the intervention group, [23,24,29,31,32,36,37,40,43,44,48,52].

Seventeen studies provided sufficient data (effect sizes and sample sizes) to be included in the quantitative synthesis. Using a random-effects model with the DerSimonian-Laird method, the pooled effect size across studies was Hedges’ g = −0.45, with a standard error of 0.045. This indicates a moderate and statistically significant effect of MBIs in reducing psychological distress. The forest plot illustrated variability in individual study outcomes, yet the overall direction of effect favored the intervention across most included trials, Figure 2.

**Figure 3:**
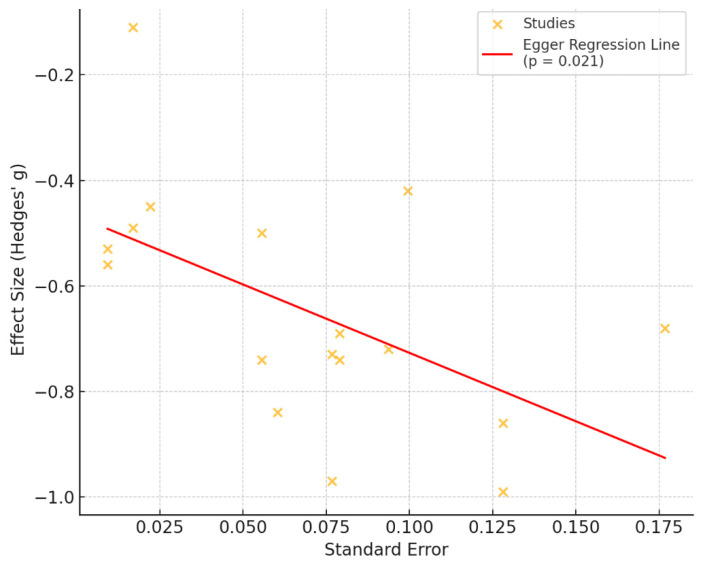
Funnel plot of effect sizes (Hedges’ g) plotted against standard error for studies included in the meta-analysis. Points represent individual studies, and the solid line shows the Egger regression line. More negative effect sizes indicate greater symptom reduction; asymmetry suggests potential publication bias (Egger’s test *p* = 0.021).

The distribution of studies showed a generally symmetrical pattern around the pooled effect estimate; however, a formal test, such as Egger’s, would be necessary to assess asymmetry. The regression test for publication bias was statistically significant (*p* = 0.021), indicating potential asymmetry in the funnel plot and suggesting publication bias in the included studies (Figure 3).

**Figure 4:**
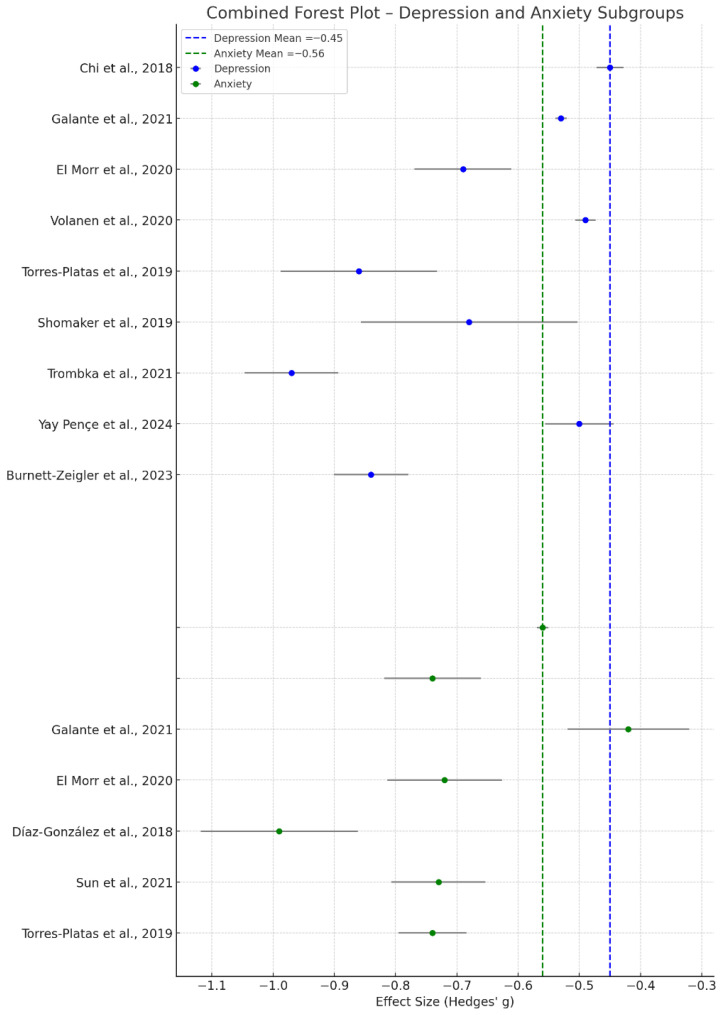
Combined forest plot of effect sizes (Hedges’ g) for depression and anxiety outcomes. Points represent study-specific estimates and horizontal lines indicate 95% confidence intervals. Dashed vertical lines indicate pooled mean effects for depression and anxiety. Negative effect sizes indicate greater symptom reduction in the intervention group, whereas positive values indicate higher symptom levels or less improvement than in the control group, [23,24,29,31,32,36,37,40,43,48,52].

Subgroup analyses were conducted to explore the differential effects of MBIs on depression and anxiety outcomes. For studies targeting depression, the pooled effect size was Hedges’ g = −0.45, indicating a moderate reduction in depressive symptoms across interventions. For studies focusing on anxiety, the pooled effect size was Hedges’ g = −0.56, suggesting a slightly more substantial effect of mindfulness practices in alleviating anxiety (Figure 4). This effect size corresponds to a small-to-moderate reduction in depressive symptoms, suggesting a clinically meaningful benefit of the intervention.

## 4. Discussion

The synthesis of 30 studies confirms that MBIs, including variations incorporating gratitude elements, are effective strategies for reducing symptoms of depression, anxiety, and stress across diverse populations and settings. Across the studies, MBIs yielded small to moderate effect sizes, with several trials of Gallo, Torres-Plates, or Trombka reporting statistically significant reductions in psychological distress in both clinical and non-clinical samples [36,37,39]. Notably, studies with university students, El Morr and Loucks [24,45], adolescents (Chi and Díaz-González) [31,40], and healthcare workers by Santamaría-Peláez [42] showed remarkably consistent improvements in emotional regulation and psychological well-being. The duration of MBIs affects their efficacy, with moderate-length programs (8–12 weeks) yielding the most reliable improvements. Shorter interventions may be insufficient for sustained change, and longer interventions, although less common, could enhance long-term psychological resilience.

Gratitude emerged as a key emotional facet cultivated through mindfulness practices, potentially enhancing the positive affect and resilience components that mediate stress-related outcomes [51,52]. Furthermore, neuroimaging studies suggest that MBIs modulate brain networks involved in emotional processing and stress regulation, reinforcing the physiological basis of their efficacy [51].

Subgroup analysis revealed that effects may be more pronounced in younger populations and in group-based, in-person formats than in digital or abbreviated versions. Additionally, studies with follow-up durations beyond eight weeks often observed attenuated effects, highlighting the importance of sustained engagement or booster sessions to maintain benefits. These findings support the utility of MBIs across both domains (depression and anxiety), with potentially higher responsiveness observed for anxiety outcomes. These differences may reflect the heightened responsiveness of anxiety symptoms to attentional and emotion-regulation strategies embedded in mindfulness practices. While individual studies varied in statistical power and methodology, the direction of effect was consistently favorable across subgroups, with nearly all studies reporting improvements from baseline.

The clinical utility of MBIs, especially those integrating gratitude-based practices, lies in their cost-effectiveness, scalability, and cultural adaptability as tools for promoting mental health. These results warrant further research into mechanism-specific pathways and the potential synergistic effects of combining mindfulness with other positive psychology constructs, such as gratitude.

Incorporating gratitude-focused practices into mindfulness protocols appears to enhance the psychological benefits of mindfulness. Studies that integrated gratitude journaling, reflective exercises, or compassion-based techniques reported greater improvements in emotional well-being, particularly in reducing depressive symptoms and enhancing self-compassion [26,52]. Gratitude may act as a synergistic emotional amplifier, reinforcing the attentional and cognitive shifts cultivated through mindfulness. The positive reframing and affective regulation facilitated by gratitude can deepen the impact of mindfulness by shifting focus from distress and rumination toward appreciation and connectedness. While only a subset of the reviewed studies explicitly addressed gratitude, the emerging pattern suggests that combining mindfulness with gratitude practices may yield more robust and sustainable improvements in mental health outcomes. Future studies should further explore this interaction by directly comparing mindfulness-alone protocols with those enriched with structured gratitude components.

Gratitude-focused interventions on their own confer only modest benefits. In the majority of included studies, gratitude was incorporated as an adjunct to mindfulness-based interventions rather than as a core, standalone component. Consequently, the reported effects likely reflect the combined impact of mindfulness and gratitude practices, which should be considered when interpreting the magnitude and specificity of the observed outcomes. A dedicated meta-analysis of gratitude exercises (27 studies) of Cregg et al. found minor pooled effects on depression and anxiety (Hedges’ g ≈ −0.25 to −0.30) [53], and a larger systematic review of 64 RCTs likewise reported that gratitude interventions lead to statistically fewer anxiety and depression symptoms than controls, but with only modest magnitude (e.g., ~7–8% reductions in symptom scores [54]. These findings suggest that while standard MBIs reliably alleviate psychological distress, adding gratitude practices may provide a slight incremental boost, particularly by enhancing positive affect and cognitive reframing, which can further ameliorate depression. Indeed, researchers have noted that acts of gratitude can serve as a valuable “therapeutic complement” to traditional mindfulness training for improving mental health [54]. Overall, the external meta-analytic evidence is consistent with the user’s results: both standard and gratitude-enriched MBIs produce significant reductions in depression, anxiety, and stress. Any added benefits from integrating gratitude tend to be subtle rather than dramatic. Still, they generally trend in the same positive direction (e.g., somewhat greater improvement in mood or well-being) rather than contradicting the effects of conventional mindfulness interventions. This convergence across reviews underscores the robustness of MBIs’ mental health benefits, while suggesting that gratitude practices may slightly augment those benefits in domains such as depressive symptoms and emotional well-being, in line with the user’s observations.

The observed heterogeneity across analyses may be partly explained by differences in study populations, intervention duration, and delivery mode (in-person versus digital). Additional sources of variability may include differences in baseline symptom severity and outcome measurement instruments across included studies.

The assessment of publication bias using Egger’s regression test indicated significant effects (*p* = 0.021), suggesting that publication bias cannot be fully excluded. The funnel plot appeared relatively symmetric, further supporting this interpretation. The risk of bias was evaluated using the Cochrane RoB 2.0 tool, which revealed that most studies had a moderate to high methodological quality, with randomization generally well reported. However, some trials lacked clear blinding or had incomplete outcome data. Overall, the evidence base was deemed robust; however, caution is warranted when interpreting results from studies with unclear or high risk in specific domains.

This review demonstrates several key strengths, including a comprehensive search strategy, a rigorous study selection process based on PRISMA guidelines, and robust statistical synthesis methods, including subgroup and sensitivity analyses. The inclusion of diverse populations across multiple countries enhances generalizability. Several methodological limitations of the included studies should be acknowledged. Many trials relied on self-reported outcome measures, which may introduce reporting and detection bias. Blinding was often not feasible due to the behavioral nature of the interventions, potentially leading to inflated effect estimates. Sample sizes varied considerably, and some studies were underpowered to detect small-to-moderate effects. In addition, heterogeneity in intervention formats, duration, and delivery modes may have contributed to variability in observed effectiveness, limiting direct comparability across studies. This review also has methodological limitations. Although a comprehensive search strategy was applied, reliance on AI-assisted platforms may have led to the omission of some relevant records that were not optimally indexed or ranked by the search algorithms. Despite independent screening and verification, the possibility of selection bias cannot be fully excluded. Furthermore, only studies reporting quantitative outcomes were included, which may have limited the inclusion of relevant qualitative insights. Finally, heterogeneity across populations, outcome measures, and intervention designs constrained the scope of subgroup analyses and limited causal inference.

The findings support the practical application of MBIs as effective tools to reduce symptoms of depression, anxiety, and stress across various age groups and settings. Integrating gratitude components appears to offer additional emotional benefits, particularly in enhancing positive affect and resilience. Clinicians and program developers may consider embedding gratitude-focused practices (e.g., journaling, reflection) more systematically within mindfulness curricula to potentially amplify outcomes. Moreover, the observed efficacy of web-based and app-supported MBIs highlights their utility for scalable, low-cost mental health care delivery.

Future studies should focus on randomized controlled trials that directly compare standard MBIs with gratitude-integrated protocols to assess potential synergistic effects. Long-term follow-up assessments are needed to determine the durability of improvements in psychological outcomes. Additionally, exploring the digital delivery of mindfulness and gratitude interventions via mobile applications or online platforms could expand access and scalability, especially for underserved or remote populations. Mechanistic studies using neuroimaging and biomarkers may also help clarify how gratitude enhances mindfulness outcomes at the psychological and physiological levels.

## 5. Conclusions

This systematic review indicates that MBIs are associated with reductions in symptoms of depression, anxiety, and stress across a range of populations and settings. In line with the study objectives, our findings indicate that mindfulness-based interventions, including gratitude-related components, are associated with meaningful improvements in depression, anxiety, and stress outcomes. The pooled effects were generally moderate, with somewhat stronger effects observed for anxiety-related outcomes. Interventions of moderate duration (8–12 weeks) tended to show the most consistent benefits. Mindfulness programs that incorporated structured gratitude components demonstrated comparable or modestly enhanced effects, particularly for emotional well-being. Although the findings are supported by acceptable risk-of-bias assessments, the heterogeneity of study populations and intervention formats warrants cautious interpretation. Overall, the results support the potential utility of MBIs in clinical and educational contexts and highlight the need for further well-designed studies to clarify the specific contribution of gratitude-integrated approaches.

## Figures and Tables

**Table 1:** Study characteristics.

Study ID	Country	Study Design	Population	Number of Participants	Intervention Type	Outcomes	Effect Sizes/Statistical Significance	Duration of Intervention	Key Findings
Chi et al., 2018 [31]	China	RCT	Adolescents/young adults (12–25), mixed clinical/nonclinical	2.042	Mindfulness-Based Stress Reduction (group)	Depression (multiple scales)	Hedges’ g = −0.45 (post), −0.24 (follow-up, not significant)	8–12 weeks	Moderate effect versus control; no significant effect at follow-up
Galante et al., 2021 [23]	US	RCT	Adults (18–73), nonclinical, global	11.605	Mindfulness-Based Programs (group)	Anxiety, depression, distress, well-being	Standardized mean difference = −0.56 (anxiety), −0.53 (depression), −0.45 (distress)	4–12 weeks	Mindfulness-Based Programs superior to no intervention; not superior to specific active controls
El Morr et al., 2020 [24]	Canada	RCT	University students (mean age 22.6)	160	Web-based mindfulness plus cognitive behavioral therapy	Depression, anxiety, stress	β = −2.21 (Depression, *p* = 0.01), β = −4.82 (anxiety, *p* = 0.006), Cohen’s d = −0.69/−0.74	8 weeks	Significant reduction in depression/anxiety
Díaz-González et al., 2018 [40]	Spain	RCT	Adolescents (13–16), clinical	101	Mindfulness-Based Stress Reduction	Anxiety, depression, stress	Effect size = 0.42 (state anxiety), *p* < 0.05	8 weeks	Significant reduction in state
Sundquist et al., 2015 [47]	Sweden	RCT	Primary care adults (20–64), clinical	215	Mindfulness group therapy	Depression, anxiety, stress	Significant within groups, not significant between groups	8 weeks	Non-inferior to treatment as usual
Sun et al., 2021 [32]	China	RCT	University students, quarantine	114	Mobile health mindfulness	Anxiety, depression	Cohen’s d = 0.72, *p* = 0.024	N/A	Superior for anxiety
Volanen et al., 2020 [43]	Finland	RCT	School children (12–15)	3.519	Mindfulness-Based Intervention (group)	Resilience, socio-emotional, depression	β = 1.18 (resilience), β = −0.49 (depression, girls)	9–26 weeks	Small effects, some subgroup benefits
Wetherell et al., 2017 [25]	US	RCT	Older adults (65+), clinical	103	Mindfulness-Based Stress Reduction (group)	Memory, worry, depression, anxiety	*p* = 0.042–0.002	8 weeks	Greater improvement in worry, depression
Torres-Platas et al., 2019 [36]	Canada	RCT	Older adults (60–85), clinical	61	Mindfulness-Based Cognitive Therapy (group)	Depression, anxiety	Cohen’s d = 0.86/0.99, *p* = 0.002/0.001	8 weeks	Large effect sizes for depression/anxiety
Loucks et al., 2021 [45]	UK	RCT	University students (18–29)	96	Mindfulness-Based College (group)	Health summary, depression, stress	*p* = 0.004 (health), 0.03 (depression)	9 weeks	Improved health summary, depression
Hoge et al., 2022 [27]	US	RCT	Adults (mean 33), anxiety disorders	276	Mindfulness-Based Stress Reduction (group)	Anxiety (Clinical Global Impression- Severity)	Change in Clinical Global Impression- Severity: −0.07, 95% CI −0.38–0.23, *p* = 0.65 (noninferior)	8 weeks	Non-inferior for anxiety
Shomaker et al., 2019 [52]	US	RCT	Adolescent girls (12–17), at-risk	32	Mindfulness (Learning to BREATHE, 6 sessions)	Depression, mindfulness	Cohen’s d = 0.68, *p* = 0.03	1 year	Reduced depression, insulin resistance
Zhang et al., 2024 [33]	China	RCT	Hypertensive adults (mean 60), clinical	60	Mindfulness-based (group)	Depression, anxiety	21.1%/17.8% reduction, *p* = 0.041	10 weeks	Significant reduction in depression/anxiety
Trombka et al., 2021 [37]	Brazil	RCT	Police officers (24–60)	170	Mindfulness-Based Health Promotion (group)	Depression, anxiety, quality of life	Cohen’s d = 0.97/0.73 (post), d = 0.60/0.51 (6 months), *p* < 0.001	8 weeks	Greater improvement in depression/anxiety
Volanen et al., 2024 [44]	Finland	RCT	School children (12–15)	3.519	Mindfulness-Based Intervention (group)	Stress, self-kindness	Cohen’s d = −0.11, *p* < 0.01–0.02	9 weeks	Small effect on stress
Antony and Prasad, 2023 [49]	India	Quasiexperimental	Nursing students	84	Mindfulness meditation (4 weeks)	Depression, anxiety, stress	*p* < 0.05	4 weeks	Significant reduction in all outcomes
Assumpção et al., 2018 [38]	Brazil	RCT	University students	48	Mindfulness training (group)	Depression, anxiety, stress	N/A	6 weeks	Protocol only
Bluth et al., 2023 [26]	US	RCT	Adolescents (mean 15.8), subsyndromal depression	59	Mindful Self-Compassion for teens (group, 8 + 6 sessions)	Depression, self-compassion	Relative risk = 2.6 (Control vs. Mindful Self-Compassion for Teens), *p* = 0.037	36 weeks	Lower risk of depression
Fort-Rocamora et al., 2024 [41]	Spain	Quasiexperimental	Adults (mean 52), clinical	128	Mindfulness (group)	Anxiety, depression	*p* < 0.001	9 weeks	Significant improvement
Hoge et al., 2018 [28]	US	RCT	Adults (mean 39), generalized anxiety disorder	72	Mindfulness-Based Stress Reduction (group)	Anxiety, stress biomarkers	*p* = 0.007–0.036	8 weeks	Greater reduction in stress biomarkers
Zhang et al., 2019 [34]	China	RCT	Adolescents, subthreshold depression	56	Mindfulness-Based Stress Reduction (group)	Depression, rumination	F = 17.721, *p* < 0.001	8 weeks	Significant reduction in depression
Simonsson et al., 2021 [46]	UK	RCT	University students	N/A	Online mindfulness	Anxiety, depression	B = −0.36, *p* = 0.025	8 weeks	Reduced anxiety
Yay Pençe et al., 2024 [48]	Turkey	RCT	Medical students (mean 22)	323	Mindfulness-Based Stress Reduction (online)	Depression, anxiety, stress	Cohen’s d = 0.50–1.03 (depression), d = 0.73–0.74 (anxiety), *p* = 0.006 (anxiety)	8 weeks	Both effective; Mindfulness-Based Stress Reduction superior for anxiety
Santamaría-Peláez et al., 2021 [42]	Spain	RCT	Healthcare professionals	112	Mindfulness-Based Stress Reduction (standard/ abbreviated)	Anxiety, depression	η2 = 0.090–0.128, *p* = 0.014–0.002	3 months	Standard Mindfulness-Based Stress Reduction effective
Gaviria et al., 2024 [51]	Switzerland	RCT	Adolescents (13–15), nonclinical	70	Mindfulness-Based Intervention (Group plus app)	Anxiety, depression, stress, functional MRI	Functional MRI/clinical, significant	8 weeks	Reduced distress, functional MRI changes
Freedenberg et al., 2016 [30]	US	RCT	Adolescents (mean 14.8), cardiac diagnoses	46	Mindfulness-Based Stress Reduction (group, six sessions)	Anxiety, depression, stress	t = 3.7, *p* = 0.001 (stress)	6 weeks	Both reduced stress
Gallo et al., 2023 [39]	Brazil	RCT	University students (mean 25)	136	Mindfulness-Based Relapse Prevention adapted Mindfulness-Based Intervention (group)	Anxiety, depression, stress, insomnia	B = 5.76 (stress), 1.55 (depression), *p* < 0.001/0.01	8 weeks	Reduced stress, depression
Wang et al., 2023 [35]	China	RCT	Adults (18–55), anxiety	150	Modified Mindfulness-Based Stress Reduction (online)	Anxiety, depression, stress	*p* < 0.001	3 weeks	Modified Mindfulness-Based Stress Reduction = Cognitive behavioral therapy, both greater than waitlist
Burnett-Zeigler et al., 2023 [29]	US	RCT	Adults (18–65), Federally Qualified Health Center, mostly Black women	274	M-Body (Mindfulness- Based Stress Reduction (group)	Depression	t = 2.14, *p* = 0.04, Cohen’s d = 0.84	8 weeks	Reduced depression
Alfurjani et al., 2023 [50]	Jordan	RCT	Nurses	195	Mindfulness-based	Stress, depression, mindfulness	F = 17.56, *p* < 0.001	4 weeks	Reduced stress, depression

## Data Availability

No new data were created or analyzed in this study.

## Supplementary Materials

The following supporting information can be downloaded at https://www.mdpi.com/article/10.3390/healthcare14050601/s1: Table S1. Excluded Studies and Reason for Exclusion; Table S2. RoB 2.0 Risk of Bias Assessment; Table S3. Extracted data for analysis. PRISMA checklist.

## Abbreviations

The following abbreviations are used in this manuscript:

MBIs	Mindfulness-Based Interventions
MBCT	Mindfulness-Based Cognitive Therapy
MBSR	Mindfulness-Based Stress Reduction
RCT	Randomized Controlled Trial
PRISMA	Preferred Reporting Items for Systematic Reviews and Meta-Analyses
RoB 2	Risk of Bias Tool version 2
PROSPERO	International Prospective Register of Systematic Reviews
