# Supplementary material for: The Effects of Mindfulness Techniques on Anxiety, Depression, and Stress, with an Emphasis on Gratitude: A Systematic Review and Meta-Analysis
Source: Healthcare (Basel). 2026 Feb 27;14(5):601. doi: 10.3390/healthcare14050601 (PMC12984879; doi:10.3390/healthcare14050601)
Supplement: Supplementary file 1 [file healthcare-14-00601-s001.zip › healthcare-4063508-PRISMA_2020_checklis.pdf]

PRISMA 2020 Checklist

Section and Topic	Item #	Checklist item	Location where item is reported
**TITLE**			
Title	1	The Effects of Mindfulness Techniques on Anxiety, Depression, and Stress, with an Emphasis on Gratitude: A Systematic Review and Meta-Analysis	Lines 2-4
**ABSTRACT**			
Abstract	2	Mental health conditions such as anxiety, depression, and stress remain among the leading global causes of disability. Mindfulness-Based Interventions (MBIs) have gained increasing attention as effective non-pharmacological strategies for reducing psychological distress. This systematic review examined 30 randomized controlled trials and quasi-experimental studies involving over 24,000 participants to evaluate the impact of MBIs on mental health outcomes, with a specific focus on the contribution of gratitude-based components. Studies varied in terms of population, duration, and format, with most demonstrating moderate to strong effects on symptom reduction, particularly in programs lasting 8 to 12 weeks. A random-effects meta-analysis was conducted, yielding a pooled effect size of Hedges' $g = -0.45$, indicating a moderate improvement in psychological outcomes. Subgroup analyses revealed slightly stronger effects for anxiety ($g = -0.56$) than depression ($g = -0.45$). Gratitude-integrated MBIs demonstrated modestly enhanced emotional benefits, suggesting a synergistic role in improving well-being. The review found low evidence of publication bias and acceptable risk of bias, supporting the moderate results. Findings underscore the value of MBIs, particularly those integrating gratitude, as scalable, cost-effective interventions in clinical and educational settings.	Lines 21-36
**INTRODUCTION**			
Rationale	3	This review was conducted to synthesize current evidence on the effects of mindfulness-based interventions on anxiety, depression, and stress, addressing an important global mental health burden. While mindfulness interventions are well studied, the specific contribution of gratitude-based components within these interventions has not been systematically evaluated. This review aims to address this gap by integrating findings across randomized and quasi-experimental studies.	Lines 40-89
Objectives	4	The objective of this systematic review was to evaluate the effectiveness of mindfulness-based interventions on anxiety, depression, and stress, with a specific focus on interventions that integrate structured gratitude components.	Lines 90-100
**METHODS**			
Eligibility criteria	5	Studies were included if they (1) involved participants aged 12 years or older; (2) evaluated multi-session mindfulness-based interventions, with or without integrated gratitude components; (3) used randomized controlled or quasi-experimental designs with a comparator group; (4) reported validated quantitative measures of anxiety, depression, or stress; and (5) provided sufficient data for effect estimation or qualitative synthesis.<br>Studies were excluded if they involved single-session interventions, purely qualitative designs, non-human populations, or lacked validated outcome measures.	Methods, section 2.2
Information sources	6	Electronic searches were conducted using the Elicit platform, accessing the Semantic Scholar corpus, PubMed, and PsycINFO. Reference lists of included studies were also screened to identify additional relevant publications.	Methods, section 2.2
Search strategy	7	Search strategies combined controlled vocabulary and free-text terms related to mindfulness, gratitude, anxiety, depression, stress, and mental health using Boolean operators (AND/OR). The full search strategy for each database is provided in the Supplementary Materials.	Methods, Section 2.2 + Supplementary Materials (Search Strategy)
Selection process	8	Two authors independently screened titles and abstracts for eligibility, followed by full-text assessment. Discrepancies were resolved through discussion and consensus.	Methods, Section 2.2
Data collection process	9	Data extraction was performed using AI-assisted tools (Elicit AI and ChatGPT) to identify relevant study characteristics and outcomes. All extracted data were independently reviewed and verified by the authors to ensure accuracy and completeness.	Methods, Section 2.2
Data items	10a	Primary outcomes included validated quantitative measures of anxiety, depression, and stress at post-intervention and follow-up time points,	Methods,

PRISMA 2020 Checklist

Section and Topic	Item #	Checklist item	Location where item is reported
		where available.	Section 2.3
	10b	Additional variables included participant demographics, intervention format, duration, delivery mode, study setting, and presence of gratitude-based components.	Methods, Section 2.3
Study risk of bias assessment	11	Risk of bias was assessed independently by two reviewers using the Cochrane Risk of Bias 2.0 tool across five domains. Disagreements were resolved by consensus.	Methods, Section 2.4
Effect measures	12	Effect sizes were reported as Hedges' g or Cohen's d for continuous outcomes, along with confidence intervals and p-values where available.	Methods, Section 2.4, 2.5
Synthesis methods	13a	Studies were considered eligible for quantitative synthesis if they reported sufficient statistical information to calculate or extract standardized effect sizes (e.g., Hedges' g or Cohen's d) for anxiety, depression, or stress outcomes. Studies lacking adequate quantitative data were included only in the narrative synthesis. Separate syntheses were conducted according to outcome domain (anxiety and depression), based on the availability and comparability of reported data.	Methods, Section 2.5 (Statistical Analysis)
	13b	When effect sizes were not directly reported, available summary statistics (e.g., means, standard deviations, test statistics, or p-values) were used to estimate standardized effect sizes where possible. Effect sizes reported using different metrics were converted to a common standardized mean difference to ensure comparability across studies. Studies with insufficient or unclear data that did not allow reliable estimation were excluded from the quantitative synthesis.	Methods, Section 2.4–2.5
	13c	Results of individual studies and syntheses were presented using structured summary tables and forest plots. Tables summarized study characteristics, outcome measures, and effect estimates, while forest plots visually displayed individual and pooled effect sizes with corresponding confidence intervals.	Results, Table 1; Figures 2–4
	13d	A random-effects meta-analysis was performed using the DerSimonian–Laird method to calculate pooled effect sizes (Hedges' g), accounting for between-study variability. Statistical analyses were conducted using Python (version 3.11) with the Statsmodels library (version 0.14.0). Statistical heterogeneity was assessed by examining the variability of effect estimates across studies and visually inspecting forest plots.	Methods, Section 2.5 (Statistical Analysis)
	13e	Potential sources of heterogeneity were explored through predefined subgroup analyses based on outcome type, with separate syntheses conducted for anxiety and depression outcomes. Differences in intervention duration, delivery format, and population characteristics were examined descriptively to aid interpretation of variability in effect sizes.	Results, Section 3.3 (Subgroup Analyses)
	13f	No formal sensitivity analyses were conducted.	-
Reporting bias assessment	14	Publication bias was assessed using funnel plots and Egger's regression test.	Methods, Section 2.5; Results, Section 3.3
Certainty assessment	15	The certainty of evidence was not formally assessed using GRADE or similar frameworks.	-
**RESULTS**			

PRISMA 2020 Checklist

<table>
<tr><th>Section and Topic</th><th>Item #</th><th>Checklist item</th><th>Location where item is reported</th></tr>
<tr><td>Study selection</td><td>16a</td><td>
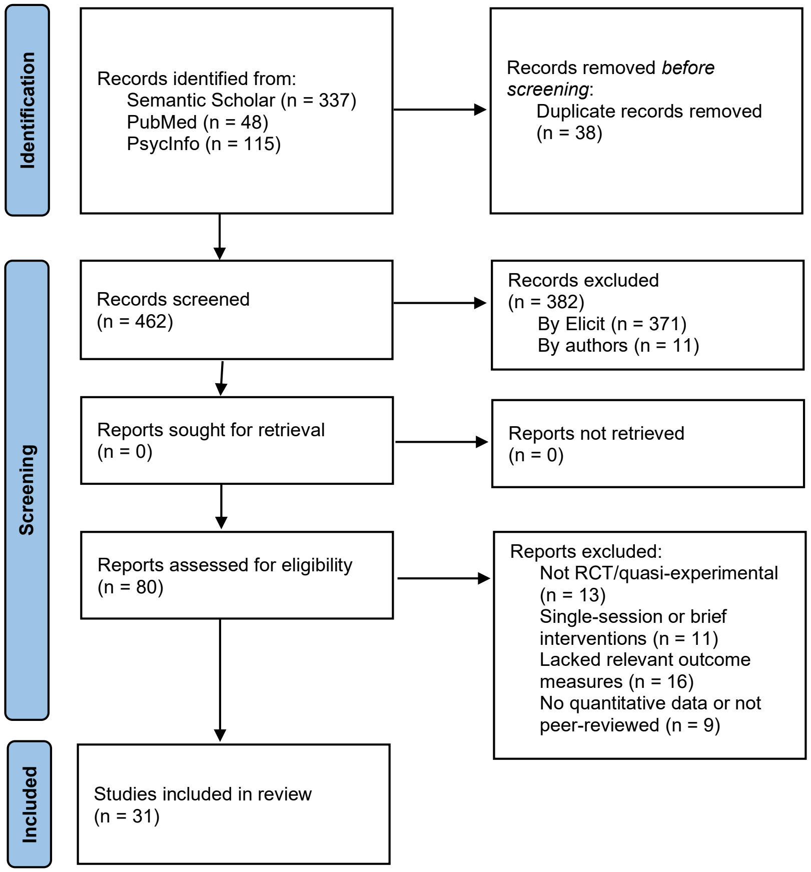

</td><td>Methods, section 2.2, Results, Section 3.1</td></tr>
<tr><td></td><td>16b</td><td>Excluded studies and reasons for exclusion are reported in Supplementary Table S1.</td><td>Supplementary Table S1</td></tr>
<tr><td>Study characteristics</td><td>17</td><td>The characteristics of the included studies are summarized in Table 1. The 30 eligible studies comprised a total of 24,563 participants and were conducted across 13 countries. Most studies employed randomized controlled trial designs, with a minority using quasi-experimental approaches. Study populations included adolescents, university students, adults, older adults, and clinical samples. Interventions varied in format, duration, and delivery mode, with most lasting between 8 and 12 weeks and using group-based mindfulness protocols.</td><td>Results, Section 3.1; Table 1</td></tr>
<tr><td>Risk of bias in studies</td><td>18</td><td>Risk of bias assessments for all included studies are presented in Table S2. Overall, 11 studies were judged to have low risk of bias, 13 showed some concerns, and six were rated as high risk. Common sources of bias included incomplete reporting of randomization procedures, lack of blinding due to the behavioral nature of interventions, and incomplete outcome data in a subset of trials.</td><td>Results, Section 3.2; Table S2</td></tr>
<tr><td>Results of individual studies</td><td>19</td><td>For each included study, outcome data and effect estimates are reported in Table 1. Most studies demonstrated statistically significant reductions in anxiety, depression, or stress following mindfulness-based interventions. Effect sizes ranged from small to large, depending on population characteristics, intervention duration, and outcome domain.</td><td>Results, Table 1</td></tr>
</table>

PRISMA 2020 Checklist

Section and Topic	Item #	Checklist item	Location where item is reported
Results of syntheses	20a	The studies contributing to the quantitative synthesis were predominantly randomized controlled trials with low to moderate risk of bias. Although heterogeneity was observed in intervention formats and populations, the direction of effects consistently favored mindfulness-based interventions across outcome domains.	Results, Section 3.3
	20b	The random-effects meta-analysis yielded a pooled effect size of Hedges' g = −0.45, indicating a moderate overall reduction in psychological distress. Subgroup analyses showed a pooled effect size of g = −0.56 for anxiety outcomes and g = −0.45 for depression outcomes, suggesting slightly stronger effects for anxiety. Forest plots illustrating individual and pooled effect estimates are presented in Figures 2–4.	Results, Section 3.3; Figures 2–4
	20c	Subgroup analyses indicated variability in effect sizes across outcome domains, with anxiety outcomes showing somewhat larger pooled effects than depression outcomes. Additional sources of heterogeneity related to intervention duration, delivery format, and population characteristics were identified descriptively.	Results, Section 3.3
	20d	No formal sensitivity analyses were conducted.	-
Reporting biases	21	Publication bias was assessed using funnel plot inspection and Egger's regression test. The results suggested the presence of potential small-study effects, indicating that publication bias cannot be fully excluded.	Results, Section 3.3; Figure 3
Certainty of evidence	22	The certainty of evidence was not formally assessed using GRADE or similar frameworks.	Discussion, Limitations subsection
**DISCUSSION**			
Discussion	23a	This systematic review indicates that mindfulness-based interventions are associated with reductions in symptoms of anxiety, depression, and stress across diverse populations and settings. The pooled effects were generally moderate, with somewhat stronger effects observed for anxiety outcomes. These findings are consistent with previous evidence supporting the effectiveness of mindfulness-based approaches as non-pharmacological interventions for mental health promotion.	Discussion, paragraphs 1–2
	23b	Several limitations of the included evidence should be acknowledged. The studies varied substantially in terms of population characteristics, intervention formats, duration, and outcome measures, contributing to heterogeneity in effect estimates. In addition, reliance on self-reported psychological measures and limited long-term follow-up in many trials may affect the precision and durability of observed effects.	Discussion, Limitations subsection (evidence-related)
	23c	This review also has methodological limitations. Although a comprehensive search strategy was used, the reliance on a limited number of databases may have led to the omission of relevant studies. In addition, the reliance on AI-assisted tools for initial data extraction, while efficiency-enhancing, may introduce the risk of oversight despite subsequent manual verification. Formal sensitivity analyses and certainty-of-evidence assessments (e.g., GRADE) were not conducted, which may limit the strength of inferences.	Discussion, Limitations subsection
	23d	From a practical perspective, the findings support the potential utility of mindfulness-based interventions as scalable and cost-effective approaches for reducing psychological distress in clinical and educational settings. The integration of structured gratitude practices may offer additional emotional benefits, although these effects appear modest. Future research should prioritize well-designed randomized controlled trials directly comparing standard mindfulness interventions with gratitude-integrated protocols, include longer follow-up periods, and explore mechanisms underlying potential synergistic effects.	Discussion, final paragraphs
**OTHER INFORMATION**			
Registration and protocol	24a	PROSPERO	-
	24b	A full review protocol was not made publicly available.	-
	24c	No amendments were made to the protocol.	-
Support	25	The study was supported by the Victor Babeș University of Medicine and Pharmacy, Timișoara, Romania, which covered the publication	Funding

PRISMA 2020 Checklist

Section and Topic	Item #	Checklist item	Location where item is reported
		costs. The funder had no role in the design, conduct, or reporting of the review.	
Competing interests	26	The authors declare no competing interests.	Conflicts of Interest
Availability of data, code and other materials	27	The extracted data is available from the corresponding authors upon reasonable request. Supplementary Materials include study selection details and risk-of-bias assessments.	Data Availability Statement; Supplementary Materials

From: Page, M.J.; McKenzie, J.E.; Bossuyt, P.M.; Boutron, I.; Hoffmann, T.C.; Mulrow, C.D.; Shamseer, L.; Tetzlaff, J.M.; Akl, E.A.; Brennan, S.E.; et al. The PRISMA 2020 Statement: An Updated Guideline for Reporting Systematic Reviews. *Syst. Rev.* **2021**, *10*, 89. https://doi.org/10.1186/s13643-021-01626-4.
