# Supplementary material for: The Effects of Mindfulness Techniques on Anxiety, Depression, and Stress, with an Emphasis on Gratitude: A Systematic Review and Meta-Analysis
Source: Healthcare (Basel). 2026 Feb 27;14(5):601. doi: 10.3390/healthcare14050601 (PMC12984879; doi:10.3390/healthcare14050601)
Supplement: Supplementary file 1 [file healthcare-14-00601-s001.zip › healthcare-4063508-supplementary.pdf]

Supplementary Materials

Table S1. Excluded Studies and Reason for Exclusion.

Study ID	Title	Reason for exclusion
Szymonik J. et al., 2024	Application of mindfulness-based interventions – MBCT and MBSR – in depression treatment and relapse prevention	Wrong study design
Jones L. et al., 2020	Mind-Body Skills Groups: A Possible Approach for Addressing Adolescent Depression in Primary Care	Wrong outcome
Areskoug E. et al., 2024	A 10-Week School-Based Mindfulness Intervention and Symptoms of Depression and Anxiety Among School Children and Adolescents: A Controlled Study	Wrong study design
Burgess E.E. et al., 2021	A Brief Mindfulness-Based Cognitive Therapy (MBCT) Intervention as a Population-Level Strategy for Anxiety and Depression	Wrong study design
Beri Y., 2024	A Comparative Study on the Impact of Gratitude on Well-Being among Youth and Adult Populations	Wrong outcome
Tan L. et al., 2015	A critical examination of the effectiveness of gratitude intervention on well-being Outcomes: A within-person experimental daily diary approach	Wrong outcome
Fang Y., 2024	A literature review addressing the relationship between mindfulness and stress-level	Wrong outcome
Franca R.D. et al. 2015	A meta-analysis of Mindfulness Based Interventions (MBIs) show that MBIs are effective in reducing acute symptoms of depression but not anxiety.	Wrong study design,
Saragih I.D. et al., 2023	A meta-analysis of mindfulness-based interventions for improving mental health and burden among caregivers of persons living with dementia.	Wrong study design,
Haukaas R.B. et al., 2018	A Randomized Controlled Trial Comparing the Attention Training Technique and Mindful Self-Compassion for Students With Symptoms of Depression and Anxiety	Wrong outcome

Table S2. RoB 2.0 Risk of Bias Assessment

Study ID	Randomization	Deviation from Intervention	Missing Outcome	Measurement of Outcome	Selective Reporting	Overall Risk
Chi et al., 2018 [31]	Some concerns	Low	Low	Moderate	Low	Some concerns
Galante et al., 2021 [23]	Low	Low	Low	Low	Low	Low
El Morr et al., 2020 [24]	Some concerns	Low	Low	Moderate	Low	Some concerns
Díaz-González et al., 2018 [40]	Some concerns	Moderate	Low	Moderate	Moderate	Moderate
Sundquist et al., 2015 [47]	Low	Moderate	Low	Moderate	Low	Moderate
Sun et al., 2021 [32]	Some concerns	Moderate	Low	Moderate	Some concerns	Moderate
Volanen et al., 2020 [43]	Low	Low	Low	Low	Low	Low

Wetherell et al., 2017 [25]	Low	Moderate	Low	Moderate	Some concerns	Moderate
Torres-Platas et al., 2019 [36]	Low	Low	Low	Low	Low	Low
Loucks et al., 2021 [45]	Low	Moderate	Low	Low	Low	Low
Hoge et al., 2022 [27]	Low	Low	Low	Low	Low	Low
Shomaker et al., 2019 [52]	Some concerns	Low	Moderate	Moderate	Moderate	Moderate
Zhang et al., 2024 [33]	Low	Low	Low	Low	Low	Low
Trombka et al., 2021 [37]	Low	Low	Low	Low	Low	Low
Volanen et al., 2024 [44]	Low	Low	Low	Low	Low	Low
Antony and Prasad, 2023 [49]	High	High	Moderate	Moderate	Moderate	High
Assumpção et al., 2018 [38]	Some concerns	Moderate	High	Moderate	High	High
Bluth et al., 2023 [26]	Low	Low	Moderate	Low	Low	Some concerns
Fort-Rocamora et al., 2024 [41]	High	Moderate	Moderate	Moderate	Moderate	High
Hoge et al., 2018 [28]	Low	Low	Low	Low	Low	Low
Zhang et al., 2019 [34]	Low	Low	Low	Low	Low	Low
Simonsson et al., 2021 [46]	Some concerns	Moderate	Moderate	Moderate	Moderate	Moderate
Yay Pençe et al., 2024 [48]	Low	Low	Low	Low	Low	Low
Santamaría-Peláez et al., 2021 [42]	Low	Moderate	Low	Moderate	Low	Moderate
Gaviria et al., 2024 [51]	Low	Moderate	Low	Moderate	Low	Moderate
Freedenberg et al., 2016 [30]	Low	Moderate	Low	Low	Moderate	Moderate
Gallo et al., 2023 [39]	Low	Moderate	Low	Moderate	Low	Moderate
Wang et al., 2023 [35]	Low	Low	Low	Low	Low	Low
Burnett-Zeigler et al., 2023 [29]	Low	Low	Low	Low	Low	Low
Alfurjani et al., 2023 [50]	Low	Moderate	Moderate	Moderate	Low	Moderate

Table S3. Extracted data for analyze

Study ID	Country	Outcome	Effect Size (Hedges' g)	p-value	Sample Size
Chi et al., 2018	China	Depression	-0.45		2042
Galante et al., 2021	US	Anxiety	-0.56		11605
El Morr et al., 2020	Canada	Depression	-0.69	0.010	160
Díaz-González et al., 2018	Spain	Anxiety	-0.42	0.050	101
Sundquist et al., 2015	Sweden	Depression			215
Sun et al., 2021	China	Anxiety	-0.72	0.024	114
Volanen et al., 2020	Finland	Depression	-0.49		3519
Wetherell et al., 2017	US	Depression		0.042	103
Torres-Platas et al., 2019	Canada	Depression	-0.86	0.002	61
Loucks et al., 2021	UK	Depression		0.030	96
Hoge et al., 2022	US	Anxiety		0.650	276
Shomaker et al., 2019	US	Depression	-0.68	0.030	32
Zhang et al., 2024	China	Depression		0.041	60
Trombka et al., 2021	Brazil	Depression	-0.97	0.001	170
Trombka et al., 2021	Brazil	Anxiety	-0.73	0.001	170
Volanen et al., 2024	Finland	Stress	-0.11	0.010	3519
Antony and Prasad, 2023	India	Depression		0.050	84
Bluth et al., 2023	US	Depression		0.037	59
Fort-Rocamora et al., 2024	Spain	Anxiety		0.001	128
Hoge et al., 2018	US	Anxiety		0.007	72
Zhang et al., 2019	China	Depression		0.001	56
Simonsson et al., 2021	UK	Anxiety		0.025	
Yay Pençe et al., 2024	Turkey	Depression	-0.5	0.006	323

Santamaría-Peláez et al., 2021	Spain	Depression		0.002	112
Gaviria et al., 2024	Switzerland	Anxiety			70
Gallo et al., 2023	Brazil	Depression		0.010	136
Wang et al., 2023	China	Depression		0.001	150
Burnett-Zeigler et al., 2023	US	Depression	-0.84	0.040	274
Alfurjani et al., 2023	Jordan	Depression		0.001	195
